# Which Is More Toxic? Evaluation of the Short-Term Toxic Effects of Chlorpyrifos and Cypermethrin on Selected Biomarkers in Common Carp (*Cyprinus carpio*, Linnaeus 1758)

**DOI:** 10.3390/toxics9060125

**Published:** 2021-05-31

**Authors:** Elenka Georgieva, Vesela Yancheva, Stela Stoyanova, Iliana Velcheva, Ilia Iliev, Tonka Vasileva, Veselin Bivolarski, Eleonora Petkova, Brigitta László, Krisztián Nyeste, László Antal

**Affiliations:** 1 Department of Developmental Biology, Faculty of Biology, Plovdiv University, 4000 Plovdiv, Bulgaria; elenkageorgieva@uni-plovdiv.bg (E.G.); stela.stoyanova@uni-plovdiv.bg (S.S.); eleonora_t_p@abv.bg (E.P.); 2 Department of Ecology and Environmental Conservation, Faculty of Biology, Plovdiv University, 4000 Plovdiv, Bulgaria; vyancheva@uni-plovdiv.bg (V.Y.); anivel@uni-plovdiv.bg (I.V.); 3 Department of Biochemistry and Microbiology, Faculty of Biology, Plovdiv University, 4000 Plovdiv, Bulgaria; iliailiev@uni-plovdiv.bg (I.I.); vasileva@uni-plovdiv.bg (T.V.); bivolarski@uni-plovdiv.bg (V.B.); 4 Department of Medical Microbiology, Faculty of Medicine, University of Debrecen, 4032 Debrecen, Hungary; laszlo.brigitta@med.unideb.hu; 5 Department of Hydrobiology, Faculty of Science and Technology, University of Debrecen, 4032 Debrecen, Hungary; antal.laszlo@science.unideb.hu

**Keywords:** pesticides, water, contamination, fish, biomarkers

## Abstract

The general aim of this study was to investigate the negative short-term effects of different concentrations of chlorpyrifos (CPF) and cypermethrin (CYP), based on the EU legislation (MAC-EQS) in common carp (*Cyprinus carpio* Linnaeus, 1758) under laboratory conditions and to compare their toxicity. The fish were exposed to the pesticides for 96 h and then different histological and biochemical biomarkers were investigated in the gills and liver, and bioaccumulation analyses were conducted. The chemical studies showed increased pesticide concentrations in the gills as the first site for pollutants compared to the liver at the 96th hour. In addition, the histological analyses showed severe alterations in the gills and liver after exposure to both tested pesticides. In the gills, we found mainly intense proliferative and, to a lesser extent, degenerative changes and alterations in the circulatory system, such as necrosis and vasodilation. In the liver, regressive and progressive lesions, as well as circulatory disturbances and inflammation, were observed. The regressive lesions showed a higher degree of expression compared to the other changes. Furthermore, we found altered enzymatic activities—catalase, glutathione reductase, and glutathione peroxidase—in the liver, compared to the control. Overall, both tested pesticides impacted the studied biomarkers in common carp, even at concentrations lower than those permitted by law. However, the results of the comparative analysis showed a relatively higher toxicity of CYP compared to CPF in the fish. Still, questions persist as to whether the observed changes are adaptive or entirely destructive. To avoid any danger or risk, these pesticides must be applied cautiously, especially near water bodies.

## 1. Introduction

Pesticides are man-made chemicals, commonly used throughout the world as plant protection products, mainly to keep crops safe from damage and enhance their yields [1]. In addition, in the last few decades they have been used on an increasingly wider scale [2]. Although pesticides play a positive role in the control of pests, their use has also been associated with a major impact on aquatic ecosystems [3,4]. According to Fevery et al. [5], their persistence in water is attributed to run-offs from agricultural fields, seepage through soil, and transportation via air.

Organophosphorus pesticides (OPs) have been used as insecticides for long periods of time, mainly after the ban of organochlorine pesticides, such as dichlorodiphenyltrichloroethane (DDT) [6]. Chronic environmental threats caused by the persistence of organochlorine and organophosphorus pesticides in ecosystems resulted in the development of new synthetic pesticides. Pyrethroid pesticides were introduced as a new class of pesticides in the 1970s, and their use has been increasing gradually with the decreasing use of the above-mentioned insecticides [7,8]. In this sense, Nunes et al. [9] explained that cypermethrin (CYP) and chlorpyrifos (CPF) are chemicals widely applied in agriculture, forestry, and the livestock industry, and the commercial mixture of CYP and CPF is frequently used, even in households.

Chlorpyrifos (CPF) is an organophosphorus (OP) insecticide with a broad spectrum of action, which inhibits the acetylcholinesterase (AChE) activity responsible for controlling the nerve impulse in the cholinergic synapses [10,11]. However, the EU announced the withdrawal of CPF without its renewal, because of its potential genotoxicity and developmental neurotoxicity. Thus, insecticides containing CPF and CPF-methyl could be marketed until 1 April 2020, and the final date of their use expired on 16 April 2020. Therefore, the mechanisms of low-dose effects of CPF as single chemicals and in mixture are still unclear, as stated by Mit et al. [12]. Nevertheless, the use of CPF outside the European Union has remained significant, which means CPF still enters aquatic environments and therefore, spreads worldwide via sea currents. For instance, CPF has been registered in Argentinean streams up to 10 mg/L in waters and 19 mg/kg in sediments [13,14] and in Indian waters between 0.019 and 2.73 μg/L [15].

Cypermethrin (CYP) is a II type pyrethroid insecticide which is considered moderately toxic [16,17]. CYP has been widely applied due to its efficacy, as the insecticide is both a stomach and contact poison, which impacts the nervous system by affecting the voltage-dependent sodium channels and inhibiting the adenosine triphosphate (ATP) enzymes; it also has low toxicity to birds and mammals. However, according to Carriquiriborde et al. [18], it is extremely toxic to aquatic organisms and must not be applied near water or when there is the possibility of a drift.

Overall, CPF and CYP are considered moderately dangerous (Class II) according to their acute toxicity. When administered orally to rats, the LD_50_ is 135 mg/kg for CPF and 250 mg/kg for CYP [19].

Aquatic organisms as non-target organisms, e.g., fish, are exposed to pesticides and their residues via different routes including run-offs or spray drifting [20]. Fish are essential components of aquatic ecosystems, playing important roles in community food web structures, nutrient recycling and productivity, as well as having high socio-economic importance [21]. In addition, fish are an important food source for humans, and monitoring the bioaccumulated toxicant levels is therefore important to ensure food safety [22]. In environmental biomonitoring and risk assessment studies, it is important to use sentinel species [23], and thus, fish have proved to be sensitive organisms for assessing the condition and functioning of aquatic ecosystems [24]. Furthermore, fish have been widely documented as useful bioindicators of environmental water quality because of their different age and trophic levels [25].

The organs of teleost fish most often investigated in toxicological studies are the gills and the liver. As fish gills are in constant contact with water, they represent an important site of waterborne toxicant uptake [26,27]. The liver is a central metabolic, detoxification, and storage organ, which has numerous anabolic and catabolic functions [28]. Moreover, in cyprinid fish the hepatopancreas, which is a cross between a liver and a pancreas, referred to as liver, is commonly studied, along with the gills, kidney, and muscles.

It is a common practice to apply biological tools for monitoring the anthropogenic impacts on aquatic wildlife [29]. According to Ballesteros et al. [30], in the multi-stressor context, contaminants can affect the structure and function of biological systems [31,32], causing responses at molecular, biochemical, histological and behavioral levels, which are known as biomarkers, before the population, community or ecosystem level is affected.

In order to reduce hazards, risks, and dependence on chemical control for plant protection, the European Union (EU) has established policies, including Integrated Pest Management (IPM) and the Water Framework Directive (WFD), for controlling pesticides and water quality [33]. The Directive sets a quality standard of 0.5 µg/L for the sum of all pesticides detected in a single sample [34]. To protect the surface and ground waters from further deterioration and to protect also the aquatic environment, the EU has issued Directive 2000/60/EC prompting every EU member to achieve ‘good ecological status’ in surface waters by 2015. Subsequently, Directive 2008/105/EC and Directive 2013/39/EU set Environmental Quality Standards (EQS) for priority substances and certain other pollutants (including 21 pesticides), which the member states should apply to surface waters. Furthermore, the European Drinking Water Directive sets a quality standard of 0.1 µg/L for each individual pesticide and its toxicologically relevant metabolite in drinking water [35,36]. We agree with Kohler & Triebskorn [37] that although the effects of pesticides on target pest organisms are well studied, clearly documented, and already known to some extent, their effects on non-target organisms are not yet fully understood.

Therefore, the general aim of the present study was to evaluate and compare the short-term harmful effects of different CPF and CYP concentrations, which were lower than those permitted on the basis of Directive 2013/39/EU, on common carp. In addition, in this study, we took into consideration for the first time the maximum allowable concentrations (MAC-EQS, 0.1 µg/L for CPF and 0.0006 µg/L for CYP), rather than the annual average ones (AA-EQS), as in our previous research [38,39]. The hypothesis underlying this work is that these concentrations will also change selected biomarkers in the fish gills and liver after a 96-h exposure under laboratory conditions.

## 2. Materials and Methods

### 2.1. Test Species

The common carp (*Cyprinus carpio* Linnaeus, 1758) is widespread and is reared in great numbers in aquaculture. For instance, the common carp is the most commonly grown freshwater fish in China, and can also serve as a bioindicator for assessing the status of environmental contamination [40,41]. Moreover, the efficacy of common carp as a sentinel species has been proven by previous studies involving laboratory experiments, field studies, or biomonitoring programs [39,42,43,44,45]. The common carp is also a bottom-dwelling species, and is hence likely to be directly exposed to toxicants via the sediment or through the consumption of contaminated benthic invertebrates [46]. However, it is relatively resilient to water pollution, which is another important characteristic of bioindicator organisms in aquatic toxicology [38,47,48,49,50].

### 2.2. Test Chemicals

**Figure 1:**
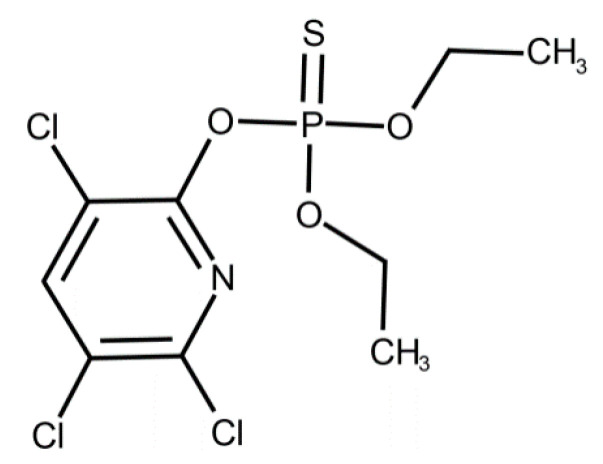
Chemical structure of chlorpyrifos. Reproduced from [51]. 2017, Elsevier.

In our study, CPF, C_9_H_11_Cl_3_NO_3_PS (*O*,*O*-Diethyl *O*-3,5,6-trichloropyridin-2-yl phosphorothioate) (CAS Number: 2921-88-2, molecular weight 350.59) was purchased from Merck (Darmstadt, Germany). The chemical structure of CPF is shown in Figure 1.

**Figure 2:**
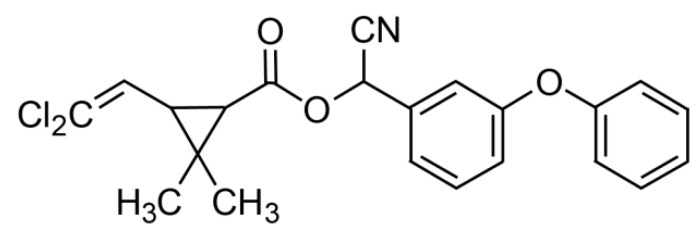
Chemical structure of cypermethrin. Reproduced from [52]. 2017, Elsevier.

Furthermore, CYP, C_22_H_19_Cl_2_NO_3_ (cyano-(3-phenoxyphenyl)methyl)3-(2,2-dichloroethenyl)-2,2-dimethylcyclopropane-1-carboxylate) (CAS Number 52315-07-8, molecular weight 416.30) was also purchased from Merck (Darmstadt, Germany). The chemical structure of CYP is shown in Figure 2.

The experimental setup was similar to that of the CPF experiment, which was described by Stoyanova et al. [39], but in the present study, we chose to test the MAC-EQC (100%, 0.1 µg/L) set in Directive 2013/39/EU, which was done for the very first time. The decreasing CPF concentrations were prepared as 50% (0.05 µg/L) and 30% (0.03 µg/L) of MAC-EQS in surface waters. CPF with a purity of 99.5% was obtained for the purpose of this study. Thus, a stock solution of 100 ppm CPF was prepared, and 100 µL of it was diluted in 900 µL methanol (10 ppm) and calculated for the tested concentrations for 50-L water tanks.

Analogously, the decreasing CYP concentrations were also based on the EU legislation (100%, 0.0006 µg/L) and prepared as 30% (0.0002 µg/L) and 50% (0.0003 µg/L) of MAC-EQS in surface waters. CYP with a purity of 97.4% was also obtained for the purpose of this study. Thus, a stock solution of 20,000 ppm CYP was prepared, 20 µL of which was diluted in 980 µL methanol (4000 ppm) and calculated for the tested concentrations for 50-L water tanks.

Overall, we applied 5, 2.5 and 1.5 µg CPF, and 0.03, 0.015 and 0.01 µg CYP for 50 L final solutions, respectively.

### 2.3. Acute Experimental Exposure

Common carp juveniles (*n* = 200), which were considered healthy, with normal morphology, and no visible alterations, were provided by the Institute of Fisheries and Aquaculture (Plovdiv, Bulgaria), where the conditions are controlled daily and any changes in the fish behavior or abiotic factors are strictly recorded and resolved. The fish were of approximately the same size [average total length 10.1 ± 0.4 (SD) cm; average body mass 11.15 ± 0.6 (SD) g]. After transportation, they were placed in a 100-L glass tank with chlorine-free (by evaporation) tap water equipped with oxygen pumps to acclimatize for a week at the Department of Ecology, Plovdiv University (Plovdiv, Bulgaria). The tank was kept under photoperiod conditions (a 12-h light: 12-h dark cycle). During the acclimatization period, the fish were fed with commercial pellets for cyprinids (CarpCo Excellent Koi Grower, Helmond, The Netherlands), but they were not fed for 2 days before the experiment started, as suggested by De Moura et al. [53]. After the acclimatization period had passed, the fish were randomly divided into seven tested groups (*n* = 15), including a control group (no added chemicals), and were treated in static conditions for 96 h (acute, short-term exposure) with nominal and hypothetically relevant concentrations of CPF and CYP [38]. No control group with methanol was used because its significantly low concentration was considered to have no observed effects [39]. The acute toxicity test was in a static environment without a change of water for 96 h. Therefore, the water was not renewed and the tested pesticides were added only at the beginning of the experiment, as suggested by other authors [54,55,56]. The experimental setup was carried out only once and as a pilot study on the negative short-term effects of CPF and CYP, supported by the National Scientific Fund (Sofia, Bulgaria).

The basic physicochemical characteristics of the tested water, including its conductivity, dissolved oxygen, pH, and temperature, were measured with a multi-parameter portable meter (MultiLine^®^ Multi 3510 IDS, WTW-Xylem Analytics, Weilheim, Germany). These characteristics were measured at the 24th, 48th, 72nd, and 96th hour during the acute, short-term experiment, as explained by the APHA (2005).

### 2.4. Dissection

Regarding the protection of animals used for scientific purposes, it was ensured that the fish were sacrificed with minimum pain, carefully following the guidelines of Directive 2010/63/EU. Prior to dissection, each fish was weighed on a scale (to the nearest 0.01 g) and measured with calipers (to the nearest 0.01 cm). Then, an anesthetic overdose was applied [100 mg/L water of tricaine methane sulfonate (MS222)] (Argent Chemical Laboratories, Redmond, WA, USA) [39] and the fish were dissected according to the protocol given by Rosseland et al. [57]. The methods were approved by the Ethics Committee at the Faculty of Biology, Plovdiv University, Bulgaria (№ 4/10.09.2019). Whole liver samples were extracted before the samples were divided for different purposes. Gill and liver samples were collected for bioaccumulation studies; gill and liver samples were also taken for histological analyses and small pieces of liver were collected for further studies on different enzymatic activities.

### 2.5. Chemical Analyses

Both water (5 batches per tested water tank) and fish samples (gills and liver from 10 fish per tested concentration) were collected at the end of the experiment, at the 96th hour, for chemical analyses of bioaccumulation. The bioaccumulation studies were carried out by experienced chemists at the regional accredited laboratory (Plovdiv, Bulgaria). The water samples were collected in dark glass containers filled to the brim in order to reduce the likelihood of oxidation and loss of acid volatile sulfide during transportation, ice-refrigerated, and transported to the laboratory on the very same day. The fish samples were rolled in aluminum foil and then collected in sterile, plastic, zippered bags and stored at −25 °C until the chemical analyses started. The samples were further homogenized, treated for saponification of fats, extracted with a mixture of organic solvents, and purified. The extracted samples of both water and biota were analyzed by gas chromatography coupled with tandem mass-spectrometry (GC-MS/MS) with an Agilent 7890B instrument (Thermo Fisher Scientific, Waltham, MA, USA). The limits of quantitation (LoQ) for CPF in water and fish were set at 0.005 µg/L and 0.15 µg/g and for CYP at 0.0002 µg/L and 0.009 µg/g wet weight, respectively.

In addition, the bioconcentration factor (BCF) was also calculated according to Mackay & Fraser [58], who defined it as the ratio of the chemical concentration in an organism C_B_, to the total chemical concentration in the water C_WT_, or to C_WD_, the freely dissolved chemical concentration in water. The BCF is expressed as follows:

BCF = C_B_/C_WT_ or C_B_/C_WD_ (1)

The authors also added that the use of C_WD_ is preferred because it only takes into account the fraction of the chemical in the water that is biologically available for uptake.

### 2.6. Method Validation

The chemicals used for bioaccumulation, histological, and biochemical analyses in this study were of analytical grade and were applied as received, without any further purification. They were purchased from Merck (Darmstadt, Germany). All plastic or glass equipment was sterile or cleaned carefully with double-distilled water prior to use. The chemical and biochemical analyses included samples in triplicates and blanks run in sequence to check for contamination, instrumental performance, peak identification, and quantification following Cui et al. [1]. The quality control (QC) and quality assurance (QA) of the chemical method were performed according to Päpke et al. [59] and Bonansea et al. [2], and included routine internal and independent external procedures. During processing of the samples certified reference materials—Chlorpyrifos D10 (CAS: 285138-81-0) and Atrazine D5 (CAS: 163165-75-1) (CPAchem, Bogomilovo, Bulgaria), as well as Cypermethrin (CAS number: 52315-07-8) (Merck, Darmstadt, Germany)—were analyzed to check the instrumental performance, peak height, and resolution. All chemical results showed a good agreement with the standards, and the recovery ranged between 95% and 101% for the water, and between 94% and 106% for the fish, respectively.

### 2.7. Selected Biomarkers

All selected biomarkers—histological alterations in the gills and liver, biochemical alterations in the liver including catalase, glutathione reductase, and glutathione peroxidase activities, as well as behavioral responses—were assessed at different departments of Plovdiv University (Plovdiv, Bulgaria). In addition to the biological tools applied, fish behavior (i.e., activity level) and physiological status (i.e., opercular beat frequency) were monitored daily, as explained by Neves et al. [60].

#### 2.7.1. Histological Analyses

The hematoxylin-eosin (H&E) staining method was applied, and all histological samples for light microscopic analysis were prepared according to Gautier [61]. For each tested concentration, the gills and liver from 10 fish were fixed in 10% neutral buffered formaldehyde. After 24 h, the preserved samples were washed in tap water and dehydrated in a series of increasing ethanol concentrations (70%, 80%, 85%, 96%, 100%, respectively). They were then cleared with xylene, infiltrated with liquid paraffin with a melting point of 54–56 °C and finally enclosed in paraffin wax. The samples were processed using a rotary microtome (Leica RM 2125 RTS, Leica Microsystems, Wetzlar, Germany). Thus, sections of 5 µm were produced and stained with H&E before they were further explored with a light microscope (Leica DM 2000 LED, Leica Microsystems, Wetzlar, Germany) connected to a digital microscope camera (Leica DM 2000 LED, Leica Microsystems, Wetzlar, Germany) for histological alterations.

The histological lesions were characterized according to the semi-quantitative system suggested by Bernet et al. [62], which we accepted for the purposes of our study, but also slightly changed. A five-degree (0–5) severity gradation scale, which represents the severity of each lesion, according to Saraiva et al. [63], was also applied. Moreover, the organ index values (I_O_) were calculated in order to classify the severity of the histological response using classes based on the scoring scheme proposed by Zimmerli et al. [64]: Class I (index < 10)—normal tissue structure with slight histological alterations; Class II (index 11–20)—normal structure with moderate histological alterations; Class III (index 21–30)—moderate modifications of normal tissue; Class IV (index 31–40)—pronounced histological alterations; Class V (index > 41)—severe histological alterations. Finally, we tried to study the prevalence of gill and liver histological alterations, which were calculated as the percentage occurrence within the total number of examined slides (*n* = 10) per fish per tested concentration.

#### 2.7.2. Biochemical Analyses

The liver samples were quickly thawed on ice and homogenized using a pyrex Potter-Elvehjem tissue grinder with PTFE pestle (Thermo Fisher Scientific, Waltham, MA, USA) in a chilled phosphate (50 mM, 300 mM NaCl, pH = 7.4) buffer. The catalase activity (CAT, EC 1.11.1.6) was determined using H_2_O_2_ as a substrate at 240 nm, following Beutler [65]. The glutathione reductase activity (GR, EC 1.8.1.7) was determined by monitoring the glutathione-dependent oxidation of NADPH at 340 nm [66]. The glutathione peroxidase (GPx, EC 1.11.1.9) was determined using the method described by Wendel [67].

All enzymatic activities were measured spectrophotometrically, using a Beckman Coulter Spectrophotometer DU 800 (Beckman Coulter, Inc., Brea, CA, USA) at 25 °C. The total protein content of the supernatant for each test was determined according to Bradford [68] with Coomassie Brilliant Blue G-250, using bovine serum albumin at an absorbance of 595 nm, and presented as milligram protein per milliliter homogenate.

### 2.8. Statistical Analyses

Past 3.03 [69] and GraphPad Prism 7 for Windows (USA) were used for statistical analysis of the data. The results from all performed analyses were expressed as mean ± standard deviation (SD). The results were presented in µg/L for the tested water and µg/g for the bioaccumulated pesticides in the tested fish organs, and U/mg protein for the CAT, GR and GPx activities. The normal distribution of data was checked with the Shapiro–Wilk test. The homogeneity of variances was tested with Levene’s test. The results were also analyzed for significance of differences among the control and treated groups of the water, gills, and liver, respectively, by one-way analysis of variance (ANOVA), followed by Tukey’s test (means comparison). The significance of results was set at *p* < 0.05.

## 3. Results

### 3.1. Physicochemical Properties of Tested Water

As shown in Table 1, the basic physicochemical properties of the water during the entire acute experiment (96 h) stayed relatively constant, without any significant or sudden changes between the control and the tested tanks (ANOVA, *p* > 0.05). Therefore, they will not be discussed further.

### 3.2. Bioaccumulation of CPF and CYP, Bioconcentration Factor (BCF)

The results on the water treated with CPF and CYP, as well as the bioaccumulated pesticides in the tested fish organs, are presented in Table 2. In the control group, CPF or CYP residues were not detected either in the water or in the fish (Table 2). It can be seen from the tables that the concentrations of CPF were generally higher than those of CYP, which is due to the higher applied tested concentrations based on the EU legislation (MAC-EQS). The CPF concentrations differed significantly between the control and the treated groups of water, gills, and liver, respectively (ANOVA, water: F = 327.8, *p* < 0.001; gills: F = 327.8, *p* < 0.01; liver: F = 14.6, *p* < 0.001) (Table 2).

There were significant differences in CPF concentrations between the control and the treated groups of water and liver (ANOVA, water: F = 137.6, *p* < 0.001; liver: F = 3.472, *p* < 0.05) (Table 2). At the same time, the CPF concentrations in the gills did not differ significantly among the different treatments (ANOVA, gills: F = 2.832, *p* = 0.08) (Table 2).

The values of the calculated BCF are also presented in Table 2. The BCF for CPF for all tested concentrations in the fish gills and liver ranged from 3 to 10, while those for CYP were above 1 only for the highest applied concentration and ranged from 0.009 to 0.1 for the lower CYP concentrations, respectively.

### 3.3. Histological Alterations

#### 3.3.1. Gills

**Figure 3:**
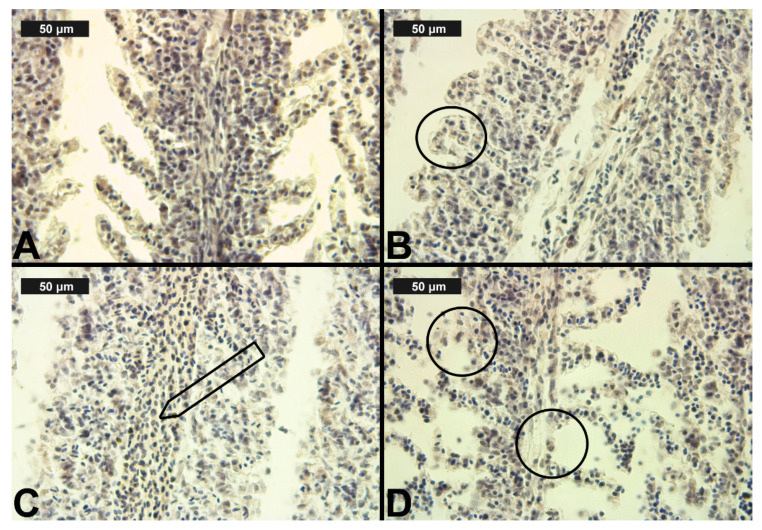
Histological alterations in the carp gills after chlorpyrifos (CPF) exposure (H&E): (**A**)—control gills; (**B**)—lamellar lifting at 0.05 μg/L; (**C**)—vasodilation of central sinus at 0.1 μg/L; (**D**)—necrosis at 0.1 μg/L CPF.

The results showed normal gill morphology in the control group. Like Laurent [70], we observed primary lamellae (gill filaments), which were closely spaced and arranged in rows. The secondary lamellae were observed across the filaments, and they were covered by a flat single-layer epithelium. Regarding the circulatory system in each lamella, we observed two main blood vessels: an afferent one, which extends from the gill arch to the tip of the filament, and an efferent blood vessel, which returns the blood to the gill arch. In terms of the scale applied, the observed control histological sections were determined as 0, despite the fact that in some individuals, we found lamellar lifting, which occupied less than 10% of the section surface. The normal histological structure of the gills of the control group of fish is shown in Table 3 and Figure 3A.

The observed histological alterations were mainly in the epithelial tissue of the gills and in the circulatory system. The degree of expression of each of the histological changes is presented in Table 3. Furthermore, the histological lesions were grouped, according to Bernet et al. [62], in three groups-lesions in the circulatory system of the organ, degenerative, and proliferative lesions (Table 3). These changes included both changes in the primary filaments and in the secondary lamellae.

After CPF exposure, a slight alteration of basal sinus vasodilation was detected in the filament at all three experimental concentrations. In contrast to the changes observed in the primary lamellae, vasodilation of the secondary lamellae was found only at the highest CPF concentration, which was expressed in a very mild degree (Table 3, Figure 3C). The degenerative changes in gill histological structure were expressed in a mild degree of necrosis of the epithelial tissue. In the filaments, necrosis was observed at both higher CPF concentrations. However, necrotic lesions concerning the secondary lamellae were detected only at the highest CPF concentration tested (Table 3, Figure 3D). The proliferative changes induced by CPF also affected both the gill filament and the secondary lamellae. We found edema in a very mild degree at all three studied CPF concentrations. The proliferation of epithelial tissue in the filament showed mainly a moderate degree of expression (3) at the higher CPF concentration applied. At the lowest tested concentration, we observed a pronounced extent of this change. Furthermore, fusion—as the most profound degree of proliferative changes—was observed at all three CPF concentrations. The degree of expression of fusion showed an increase with increasing concentrations of the pesticide. Therefore, at the lowest CPF concentration, we noticed a very mild degree of fusion, while at the higher concentrations, the degree of fusion was moderate and severe, respectively. Glandular cell proliferation of the filament was not seen after the present CPF exposure. The proliferative changes concerning the secondary lamellae were expressed in lamellar lifting and proliferation of the epithelium. Lamellar lifting showed a pronounced expression at all three experimental concentrations of CPF. Proliferative changes in the epithelial tissue were observed to a moderate extent at the lower concentrations, while at the highest concentration, they were expressed to a very mild extent (Table 3, Figure 3).

**Figure 4:**
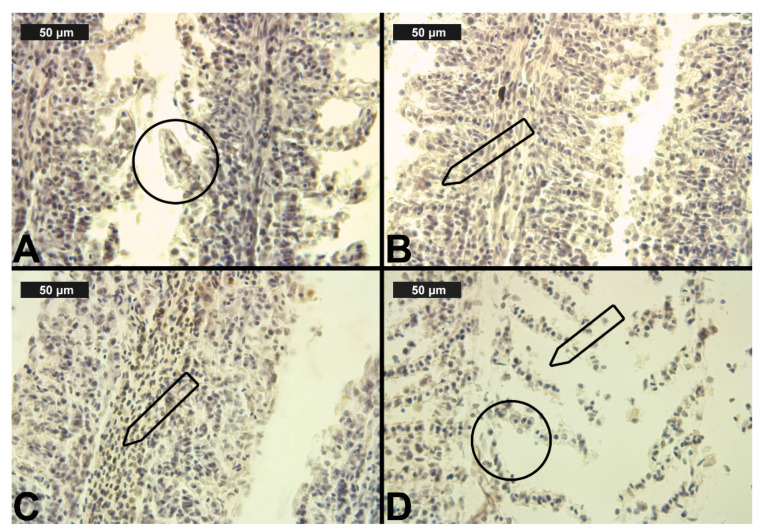
Histological alterations in the carp gills after cypermethrin (CYP) exposure (H&E): (**A**)—lamellar lifting at 0.0002 μg/L; (**B**)—epithelial proliferation at 0.0003 μg/L; (**C**)—vasodilation of central sinus at 0.0006 μg/L; (**D**)—necrosis at 0.0006 μg/L CYP.

In terms of CYP exposure, we observed changes in the circulatory system of the gills, which were expressed in vasodilation. The vasodilation detected in the secondary lamellae was very slight at all three CYP concentrations. In the filament area, vasodilation was found to be very mild at the lower concentrations, and it was described as moderate only at the highest CYP concentration (Table 3, Figure 4C). The necrotic changes were expressed mainly in a mild degree, and only at the highest concentration of exposure did they reach a moderate degree (Table 4, Figure 4D). As in the case of CPF treatment, the proliferative changes in the gills were observed in both the filament and the secondary lamellae. After 96 h of exposure, edema was found to be very mild at all three experimental concentrations. Epithelial cell proliferation in the filament was expressed to a greater extent at all three experimental concentrations (see Table 3, Figure 4B). Furthermore, fusion was present in a pronounced degree at all CYP concentrations. As with CPF, glandular cell proliferation was not detected: although this change was recorded in single sections, it affected less than 10% of the gill surface. The proliferative changes in the secondary lamellae were expressed in lamellar lifting and proliferation of the gill epithelium, respectively, in a mild and moderate degree at all applied CYP concentrations (Table 3, Figure 4A).

Comparing the indices of histological changes in the circulatory system (I_GC_), the highest value was calculated for the pesticide concentrations representing the MAC-EQS in water. For CPF, I_GC_ was 3, while for CYP I_GC_ was higher (=5). This indicates that CYP has a greater effect on the degree of expression of the changes in the circulatory system of the organ, which proves that it has a more severe effect on the structure of the organ (Table 3). Regarding the indices for degenerative changes (I_GR_), higher values were also calculated for the higher pesticide concentrations tested. Similar to the changes in the circulatory system, for CYP exposure we found higher values than CPF (I_GR_ = 3), I_GR_ = 6 was calculated for the highest applied CYP concentrations (Table 3). The indices for proliferative changes (I_GP_) after CPF exposure showed a tendency to increase with respect to the increasing pesticide concentration to I_GP_ = 22. For CYP exposure, I_GP_ = 19 was calculated for all experimental concentrations (Table 3).

According to the scheme proposed by Zimmerli et al. [64] and our results on CPF exposure, the calculated gill index falls into the Class II group only for the lowest CPF concentration—a normal histological structure with moderate pathological changes (reversible alterations). The other two concentrations fall into Class III (index 21–30)—moderate pathological changes in the histological structure (reversible alterations). In the case of CYP exposure, all three experimental concentrations led to histological changes belonging to Class III (index 21–30)—moderate changes in the histological structure (reversible alterations), which again confirms the higher toxicity of CYP compared to CPF.

#### 3.3.2. Liver

**Figure 5:**
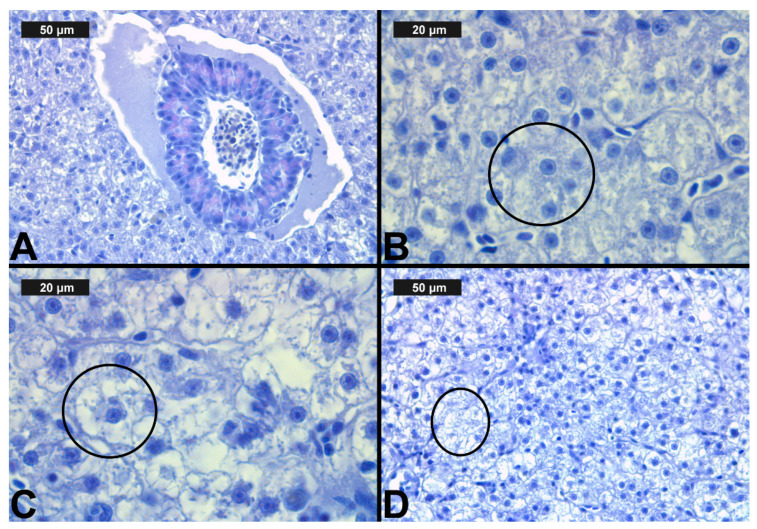
Histological alterations in the carp liver after chlorpyrifos (CPF) exposure (H&E): (**A**)-control group; (**B**)-granular degeneration at 0.05 μg/L; (**C**)-vacuolar degeneration at 0.1 μg/L; (**D**)-necrosis at 0.1 μg/L CPF.

In the control group, we observed relatively normal liver morphology (Table 4, Figure 5A). The hepatic structure of the control fish was as described by Hundet & Prabhat [71]. Furthermore, it was characterized by compactly arranged hepatocytes disposed in a simple layer aligned with sinusoids. The parenchyma itself was primarily composed of polyhedral hepatocytes, typically with central nuclei with densely stained chromatin margins and a prominent nucleolus. We also observed the pancreatic mass, which was situated around the branches of the hepatic portal veins. In addition, the pancreatic mass consisted of two parts, exocrine and endocrine. The exocrine cells were larger and elongated, while the endocrine cells were smaller and round. The morphology of the exocrine cells showed that they were arranged in an acinus form with a distinct nucleus, while the endocrine cells were scattered in between the hepatic portal veins and the exocrine pancreas. Furthermore, the venous blood entered the liver caudally from the intestine via the hepatic portal veins and branches into capillaries; the sinusoids were lined with reticuloendothelial cells, which were in turn surrounded by hepatocytes [71].

**Figure 6:**
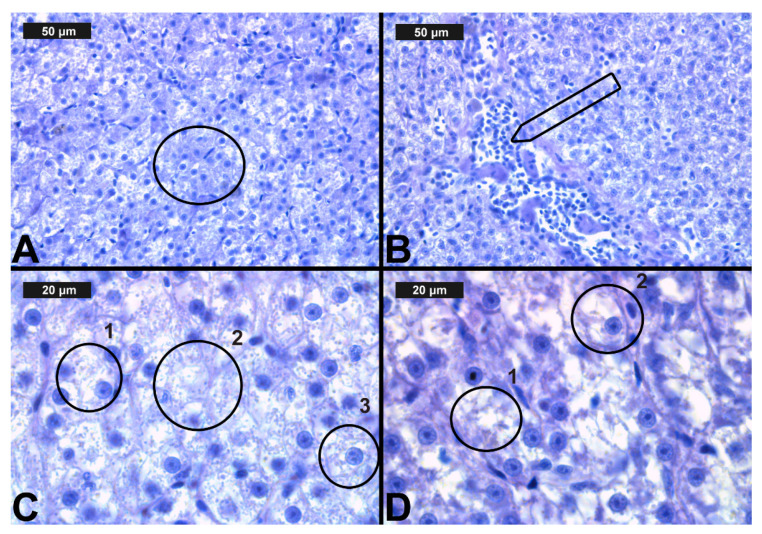
Histological alterations in the carp liver after cypermethrin (CYP) exposure (H&E): (**A**)—granular degeneration at 0.0002 μg/L; (**B**)—hyperemia at 0.0003 μg/L; (**C**)—lipid deposits (1), karyolysis (2) and vacuolar degeneration (3) in the hepatocytes (3) at 0.0006 μg/L; (**D**)—necrosis (1) and vacuolar degeneration (2) at 0.0006 μg/L CYP.

In regard to our results and the semi-quantitative system of Bernet et al. [62], we categorized the histological alterations in the liver into four main groups: circulatory, regressive, progressive, including inflammation (Table 4, Figure 5 and Figure 6).

The regressive liver lesions due to CPF exposure, i.e., granular and vacuolar degeneration, were expressed at the highest degree (see Figure 5B,C). As shown in Table 4, these changes showed a tendency towards an increase in the degree of expression with increasing CPF concentrations. With regard to the fatty degeneration, we found this alteration to be of a mild degree at the higher CPF concentrations, showing lipid accumulation in the cytoplasm of the hepatocytes. Necrobiotic changes, such as karyolysis, karyorrhexis, and karyopyknosis, were found mainly in a mild degree of expression. In addition, we also found necrosis of a moderate degree at the highest CPF concentration (Figure 5D). In regard to the progressive alterations in the liver, we observed hypertrophy, which was expressed in a moderate degree at the highest CPF concentrations, while at the other two, lower experimental concentrations, this histological change was expressed in a mild degree. The lesions that occurred in the circulatory system were expressed on the one hand in hyperemia (Table 4), the grade of which was determined by the proposed assessment scale as moderate at the higher tested concentrations, while at the lowest concentration this lesion was present in a mild degree (Table 4). On the other hand, we found inflammation, which was expressed as lymphocytic proliferation of a mild degree, at all tested CPF concentrations.

The regressive lesions due to CYP exposure (Table 4), i.e., granular (Figure 6A) and vacuolar degeneration (Figure 6C), were found at the highest degree of expression compared to CPF exposure. Moreover, the degree of granular degeneration decreased, while the expression of vacuolar degeneration increased with increasing CYP concentrations. We also observed lipid deposits (see Figure 6C) in the cell cytoplasm, mainly in a mild degree. Nuclear alterations and necrosis were also present, mainly in a mild degree (Figure 6D). The expression was found to be moderate only at the highest CYP concentration. The circulatory disturbances in the liver due to the CYP exposure were presented as hyperemia (Figure 6B). We observed this alteration in a mild degree at the lowest CYP concentration, while at the higher CYP concentrations, we found pronounced hyperemia. Moreover, lymphocytic infiltration was seen in the liver, showing a tendency to increase in its degree of expression with increasing CYP concentrations (Figure 6C). The progressive lesions were manifested as moderate hypertrophy of liver cells.

Concerning the histological changes in the circulatory system (I_LC_), the index of circulatory disturbances after CPF exposure was lower (I_LC_ = 2) than after CYP exposure (I_LC_ = 3). In regard to the regressive lesions, the index for CPF (I_LR_ = 22) was higher compared to CYP (I_LR_ = 22). However, the overall organ index (I_O_) was higher for CYP (Table 4), because after CYP exposure we detected more severe lesions, such as inflammation.

In terms of the overall liver index, at the lowest CPF concentration, we found Class I (index = 10)—normal tissue structure and slight histological alterations. The liver index at the middle-tested concentration was categorized as Class II (index 11–20)—normal structure with moderate histological alterations, while the highest concentration showed Class III (index 21–30)—moderate modifications of normal tissue. The organ index for CYP was categorized as follows: Class II (index 11–20)—moderate modifications of normal tissue at the lowest CYP concentration, while at the middle concentration, it was categorized as Class III (index 21–30)—moderate modifications of normal tissue, and at the highest CYP concentration as Class IV (index 31–40)—pronounced histological alterations, respectively.

### 3.4. Biochemical Alterations

The changes in the activity of the tested enzymes after CPF and CYP intoxication varied with the different concentrations.

**Figure 7:**
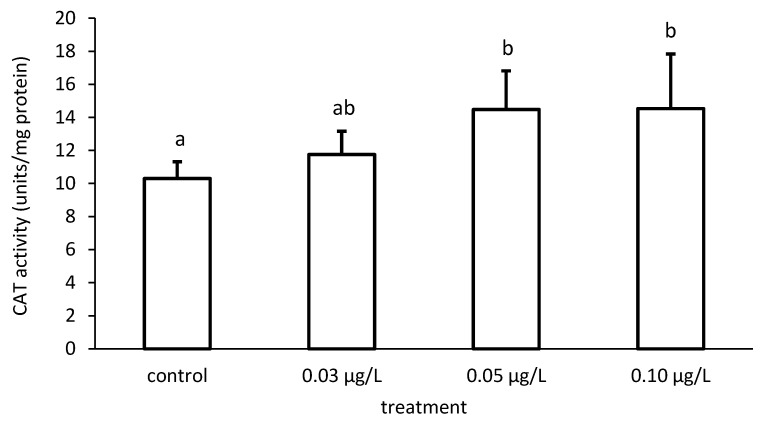
Catalase (CAT) activity in the liver of common carp under different chlorpyrifos (CPF) exposures. Bars represent the means ± SD of the control and experimental groups, measured at the 96th hour. Different letters indicate significant differences among treatments (*p* < 0.05).

**Figure 8:**
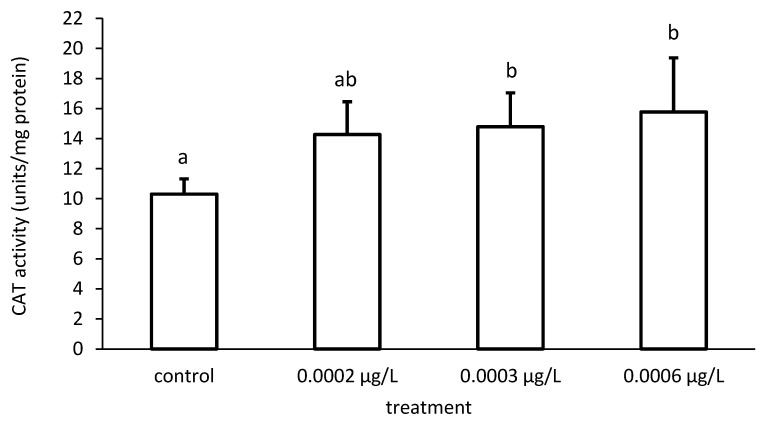
Catalase (CAT) activity in the liver of common carp under different cypermethrin (CYP) exposures. Bars represent the means ± SD of the control and experimental groups, measured at the 96th hour. Different letters indicate significant differences among treatments (*p* < 0.05).

The activity of CAT increased compared to the control, depending on the applied CPF concentrations (Figure 7 and Figure 8). In addition, the CAT activity differed significantly among the different groups (ANOVA F = 4.51; *p* < 0.05) (Figure 7).

Furthermore, the activity of CAT also increased proportionately to the increase in CYP concentrations (Figure 7). There were significant differences among the different groups in CAT activity (ANOVA F = 4.86; *p* < 0.05) (Figure 8).

**Figure 9:**
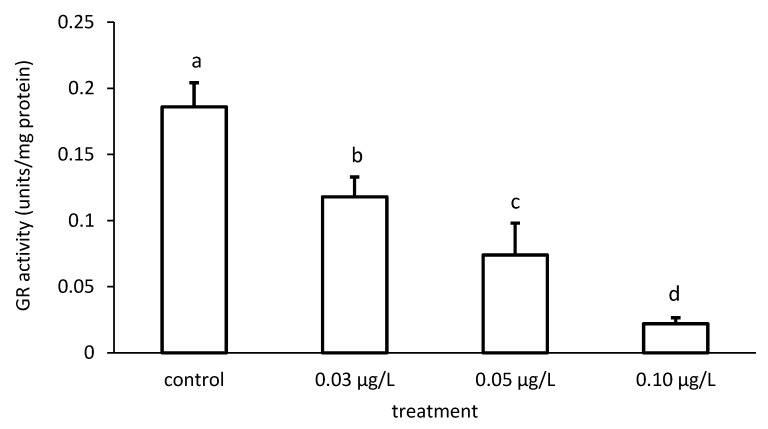
Glutathione reductase (GR) activity in the liver of common carp under different chlorpyrifos (CPF) exposures. Bars represent the means ± SD of the control and experimental groups, measured at the 96th hour. Different letters indicate significant differences among treatments (*p* < 0.05).

**Figure 10:**
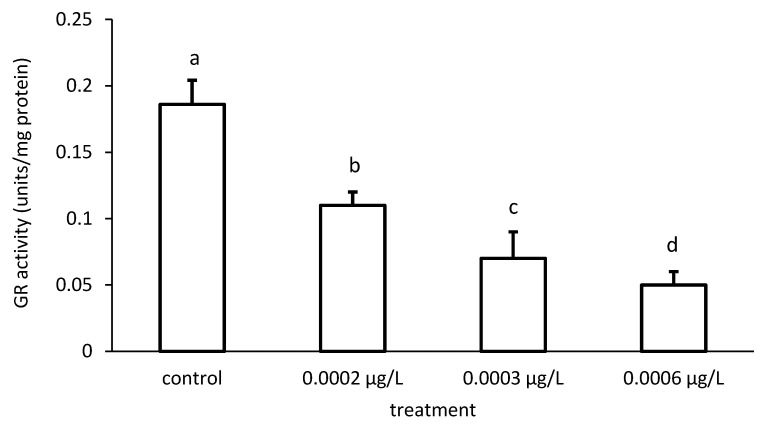
Glutathione reductase (GR) activity in the liver of common carp under different cypermethrin (CYP) exposures. Bars represent the means ± SD of the control and experimental groups, measured at the 96th hour. Different letters indicate significant differences among treatments (*p* < 0.05).

The activity of GR was reduced compared to the control, depending on the applied CPF and CYP concentrations (Figure 9 and Figure 10), respectively. The GR specific activity differed significantly among the different groups treated with both chemicals (CPF: ANOVA F = 83.94; *p* < 0.001; CYP: ANOVA F = 77.65; *p* < 0.05).

**Figure 11:**
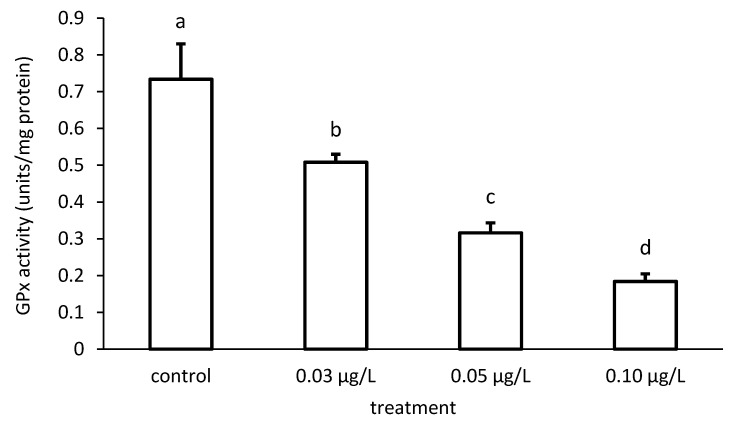
Glutathione peroxidase (GPx) activity in the liver of common carp under different chlorpyrifos (CPF) exposures. Bars represent the means ± SD of the control and experimental groups, measured at the 96th hour. Different letters indicate significant differences among treatments (*p* < 0.05).

Like that of GR, the activity of GPx was reduced compared to the control, depending on the applied CPF concentrations (Figure 11). The specific GPx activities of the groups differed significantly in the case of the CPF treatment (ANOVA F = 83.94; *p* < 0.001).

**Figure 12:**
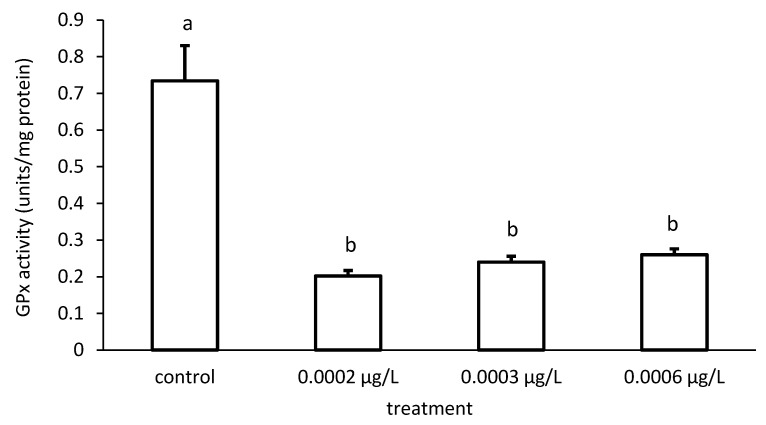
Glutathione peroxidase (GPx) activity in the liver of common carp under different cypermethrin (CYP) exposures. Bars represent the means ± SD of the control and experimental groups, measured at the 96th hour. Different letters indicate significant differences among treatments (*p* < 0.05).

In addition, the specific GPx activities of the groups also differed significantly in the case of CYP exposure (ANOVA F = 126.8; *p* < 0.001) (Figure 12).

### 3.5. Behavioral Responses

The control group showed normal behavior during the 96-h exposure period. The changes in the behavioral responses of the fish exposed to CPF and CYP fish began on the first day after dosing. The fish treated with the lower CPF (0.05 and 0.03 µg/L) and CYP concentrations (0.0003 and 0.0002 µg/L), which equaled 50% and 30% of the MAC-EQS, showed a behavior similar to that of the control group. However, the fish exposed to the highest tested CPF (0.1 µg/L) and CYP (0.0006 µg/L) concentrations, which equaled 100% of the MAC-EQS, showed behavioral changes due to toxicity, such as vertical hanging in the water, loss of equilibrium and erratic swimming, widening of the mouth, operculum, and quick gill movement. Even though the fish were obviously stressed, no mortality was recorded during the entire acute experiment.

## 4. Discussion

### 4.1. Bioaccumulation

Our results on the bioaccumulation of both tested pesticides are consistent with the findings of previously published experiments [72,73] using static exposure systems, where the reduction of the concentration of waterborne pesticides, such as CYP, was mainly attributed to the bioaccumulation and metabolism of the toxicant. Moreover, according to Michelangeli et al. [74], synthetic pyrethroid insecticides are known to be more hydrophobic than other classes of insecticides.

As explained by Glickman & Lech [75], the sensitivity of fish to pesticides such as organophosphorus chemicals and pyrethroids is thought to be due to the slow metabolism of these compounds.

According to Mackay & Fraser [58], the process of bioconcentration in fish involves the uptake of chemicals by absorption from the water only (usually under laboratory conditions). This can occur via the respiratory surface or the skin, and thereby results in the concentration of the chemical in the fish body being higher than that in ambient water.

We agree with Olisah et al. [76] that the process of bioconcentration should be viewed as one of the predominant routes of accumulation of organic contaminants in fish gills. They appear to accumulate high levels of pesticides through the mechanism of absorption due to their large surface area, since they are in direct in contact with water (representing 50% of the surface area in a fish) [77]. Our results show that the gills accumulated more pesticides by the 96th hour than the liver, which we associate with the specificity of the acute exposure and the initial stress provoked by the toxicants tested. In our study, we also confirmed the findings of Datta & Kaviraj [78], that pesticides, owing to their properties, are easily absorbed, even at low concentrations, via fish gills. Furthermore, we confirmed the results of Viran et al. [79] that pyrethroid insecticides, due to their more lipophilic character, have a high rate of gill absorption even at low concentrations, which leads to higher toxicity in fish.

In addition, we consider that evaporation is another key factor in influencing the concentrations of CPF and CYP measured in the present study. The rate of evaporation from water surfaces is expected to be reduced due to adsorption of the pesticide on the suspended matter and sediment in the aquatic environment. For instance, in water at pH 7.0 and 25 °C, the half-life of CYP is determined to be between 35 and 78 days [80,81]. According to Haya [74] and Sprague [75], temperature is also an important factor modifying the toxicity of contaminants, and it has been proven that the toxicity of pyrethroids to fish depends on the water temperature. However, we did not find any sudden increase in water temperature compared to the control, as it remained relatively constant throughout the experiment. Thus, we consider that in the present study the toxic character of the tested pesticides and the applied concentrations were the main factors responsible for the negative effects seen in common carp.

We agree with Wassenaar et al. [82] that the fish BCF is an important aspect within bioaccumulation assessments. Based on our calculations, and according to Nikanorov et al. [83], we can regard the fish used in the present study as macroconcentrators for CPF (BCF > 2) and for the highest tested CYP concentration. However, the fish can be categorized as microconcentrators (1–2) and deconcentrators (<1) for the lower CYP concentrations. Even though the BCF values for CYP were relatively low in both the gills and the liver (Table 3), we consider that the alterations observed in the studied biomarkers were triggered by the tested pesticides and their extremely toxic character, particularly that of CYP. Another important characteristic is the bioaccumulation potential of chemicals. In this sense, our results prove that despite the lower BCFs compared to, for example, heavy metals, the applied pesticide concentrations, lower than those allowed in Directive 2013/39/EU, could lead to changes in different measures of the biological status of fish. According to the US EPA [84], CPF has the potential to bioaccumulate in different tissues of aquatic species and Racke [85], who exposed various fish species to CPF continuously during early development, had calculated BCF values (ranging from 58 to 5100) many times higher than we did in our study.

Most EU countries have not established their permitted levels of toxicants regarding the health of aquatic life. In addition, unification of these concentrations within the EU members seems to be difficult to achieve. However, according to the Canadian Water Quality Guidelines [86], the CPF concentrations established for the protection of biota in freshwater is 3.5 ng/L. Moreover, according to the Argentinean Environmental Water Quality Guidelines (Niveles Guía Nacionales de Calidad de Agua Ambiente) [87], the limit of alpha-CYP for the protection of aquatic biota is only 0.6 ng/L. Even though the allowable concentrations of CPF and CYP in surface waters are higher according to the EU law regarding the quality of freshwater, we showed that the changes in the tested biomarkers could be provoked by even lower levels of these chemicals. Therefore, we believe that careful consideration should be given to reducing the currently accepted concentrations of these pesticides in waters, and each member state should urgently start working on proposing its own permissible toxicant concentrations in fish regarding possible changes in complex biomarkers. However, it is an important milestone that the marketing and use of insecticides containing CPF have been banned in the EU since April 2020.

### 4.2. Histology

Summarizing the results of the comparative histological study of CPF and CYP, we found that CYP, although its applied concentrations were lower than those of CPF, had a more severe toxic effect on the gill structure than on the liver of common carp. Only at the highest CYP concentration did we observe severe effects on the liver due to the higher degree of regressive lesions and inflammation.

Overall, we confirmed the results of Viran et al. [79] who state that, due to their lipophilicity, organic compounds such as pesticides have a high rate of absorption through the gills even when they are present in the water at very low concentrations. This in turn is a contributory factor to the sensitivity of fish to waterborne pesticide exposure, because fish do not seem to be able to metabolize these chemicals properly. According to Caliskan et al. [88], pesticides are highly toxic to fish gills, leading to severe changes in the epithelium, which impair the gaseous exchange. Moreover, our results are in line with the findings of Das & Mukherjee [89], that the pesticides bioaccumulated via the gills subsequently lead to alterations in the liver of fish.

We agree with Deb & Das [90] in that the histological changes observed in the fish gills can be used as reliable biomarkers to assess the impact of pesticides in freshwater ecosystems. We also agree with Wenderlaar Bonga & Lock [91], who accept that fish gills are a mirror image of water quality. Concurring the opinion of Camargo & Martinez [92], we consider that by determining the degree of expression of histological changes in fish gills, given their participation in respiration, osmoregulation and excretion, the level of impact of the applied toxicants can also be traced and determined.

Our results on gill histology are similar to those reported by Macirella et al. [93] and Khan et al. [94], who studied the effects of CPF and CYP, respectively. Our opinion is in line with that of Schwaiger et al. [95] that histological changes in the circulatory system suggest more pronounced stress in the fish exposed to the applied toxicants. Overall, the changes in the circulatory system were more pronounced in the main sinus in the filament. A higher degree of expression of these alterations was found at the highest concentration of CYP, which is an indicator of a stronger toxic effect in the fish body compared to that provoked by CPF. Another biomarker for the toxic effect of the applied pesticides is the presence of degenerative changes in the gill epithelium. The gill epithelium occupies a dominant position in terms of gas exchange, ion regulation, maintenance of acid-base balance, and nitrogenous waste. Thus, gill functioning is vital for fish. The degenerative alterations were expressed mainly to a mild degree, which is an indicator of the initial stage of necrotic processes in the epithelial tissue of fish gills. Furthermore, degenerative processes were found mainly at the middle and the highest pesticide concentrations. Similar degenerative changes were found by Theurkar et al. [96] and Rose et al. [97], who observed degeneration and necrosis of the epithelial cells covering the filament after sublethal insecticide concentrations. The proliferative changes were generally more typically manifested in the gill epithelium compared to the other histological alterations. Like Arellano et al. [98], we consider that lamellar lifting and edema are the first signs of proliferative changes, and can serve as a protective mechanism because the separation of the epithelium from the lamellae increases the distance over which the waterborne contaminants must pass to reach the bloodstream. In addition, we think that these mechanisms build an additional barrier between the pollutants and the gas exchange. This slows down the flow of the toxicant, which most likely activates other compensatory—adaptive mechanisms. An observation worth mentioning is that a higher degree of expression of lamellar lifting was found after CPF exposure. At the lowest concentration of CPF, a higher degree of proliferation of the gill epithelium was found, while fusion showed a very mild degree. This is an indicator of the involvement of compensatory—adaptive mechanisms in the fish organism. At the middle concentration the degree of the two changes equalized, while only at the highest concentration was fusion expressed in a higher degree. At that concentration, the structure of the filaments was completely altered, which was indicative of enhanced compensatory—adaptive mechanisms, due to the negative action of the toxicant. In contrast, after CYP exposure, the degree of expression was maintained at all three experimental concentrations, which was an indication of active cell division processes. Like us, Cengiz [99], Ayandiran et al. [100], Butchiram et al. [101], and Thayappan et al. [102], also found such histological lesions as a result of the activation of epithelial cell division processes.

We agree with Boran et al. [103] and Nataraj et al. [104], who consider that the state of fish liver morphology could serve as an indicator of chemical toxicity. Our results are in agreement with those obtained by Ghayyur et al. [105] in regard to fish exposed to pesticides (blood congestion, lymphocytic infiltration, pyknotic nuclei, necrosis, blood sinusoid dilation, vacuolation and hypertrophy). However, we found significant vacuolar degeneration. Our results also showed a higher degree of expression of lipid degeneration after CYP exposure. According to Oliveira Ribeiro et al. [106] and Vieira et al. [107], cell vacuolization occurs when the cell metabolism is severely altered by chemical stress. We agree that the accumulation of lipids in vesicles constitutes a mechanism of cellular response to the presence of lipophilic chemical agents, where this accumulation represents an attempt to immobilize these substances, preventing their interaction with other cellular components and, in this way, minimizes the toxic effect. Regarding the degree of expression of liver changes after both exposures, we found that CYP led to a higher toxicity compared to CPF, which was mainly expressed in a higher level of circulatory disturbances, inflammation, and necrotic changes.

The histological alterations found in the liver of common carp suggest that the fish exposed to CPF and CYP probably experienced a metabolic crisis caused by severe tissue damage, which was confirmed by the activities of the hepatic enzymes analyzed.

### 4.3. Biochemistry

It is a well-known fact that the metabolism of pesticides generates reactive oxygen species (ROS) in fish tissues [108]. In addition, oxidative stress plays an important role in the toxicity of different groups of pesticides, such as organophosphorus [109] and pyrethroid insecticides [110]. However, fish have a defense system, which includes antioxidant enzymes, protecting them against the harmful effects of free radicals. According to Wu et al. [111] and Yang et al. [8], such enzymes are catalase (CAT), the glutathione system itself and superoxide dismutase (SOD). According to Üner et al. [112], among the antioxidant enzymes, SOD and CAT are considered the vital first-line defenses against oxidative stress.

CAT is distributed widely in the fish tissues and is involved in the decomposition of hydrogen peroxide (H_2_O_2_) produced from SOD activity [113]. However, the reduction in CAT and GP_X_ activity leads to the accumulation of H_2_O_2_ and enhances lipid peroxidation, as explained by Halliwell & Gutteridge [114]. On the other hand, the enhanced activity of CAT is attributed to an increase in the substrate concentration, resulting in the maintenance of the H_2_O_2_ level, and this is an adaptive mechanism against oxidative damage, as stated by Liu et al. [115].

The glutathione system plays an important role in regulating the redox state of the cell, and it comprises glutathione (GSH and GSSG), glutathione peroxidase (GPx), glutathione S-transferases (GST), and glutathione reductase (GR) [116]. In addition, GR is an enzyme necessary for the reduction of glutathione disulfide (GSSG) to glutathione (GSH) and it is required to protect the cells from oxidative stress in fish [117].

GPx can catalyze GSH to reduce the lipid peroxides to harmless alcohols in order to prevent lipid peroxidation and to protect the integrity of cells from oxidative damage [118,119]. According to Gabbianelli et al. [120], GPx is also responsible for catalyzing the transformation of lipid hydroperoxides to less reactive species. We agree with Zhou et al. [66] that a decrease in the GPx activity could possibly be associated with a decrease in the protection against oxidative stress.

SOD catalyzes the dismutation of the superoxide anion radical to H_2_O and H_2_O_2_, which is detoxified by both CAT and GPx activity. Due to the inhibitory effects on oxyradical formation, the SOD–CAT system provides the first line of defense against oxygen toxicity [121] and is generally used as a biomarker indicating ROS production [122,123]. Increases in these enzyme activities are probably a response to increased ROS generation due to pesticide toxicity [124]. Our biochemical results showed an increase in CAT activity. According to the opinion of Łukaszewicz-Hussain & Moniuszko-Jakoniuk [125], which we support, the increase in hepatic CAT activity could be explained as a response of the liver to high levels of H_2_O_2_, an idea which we also support. Such an increase in the activities of SOD and CAT has been observed in *C. denticulata sinensis* [126], *L. rohita* [127] and *U. gibbus* [128].

The activity of GPx and GR in the tested fish can decrease after exposure to xenobiotics, as reported by Livingstone [129]. In our study the specific enzymatic activity of GPx and GR decreased compared to the control fish. Such inhibition of these enzymes was also found by Narra [130]. In agreement with Cheung et al. [131], we suggest that the decreased GPx activity was accompanied by decreases in the GSH levels. Using GSH as a reducing agent, the GPx enzymes catalyze the reduction of H_2_O_2_ and organic peroxides to water and their corresponding stable alcohols. The GPx activity depends on the presence of GSH, which is oxidized in this process. Thus, the GPx activity is likely to be influenced by GSH levels. We agree with Cheung et al. [131] who stated that the decreased GPx activity may also be related to the decreased availability of GSH needed to reduce the impact of ROS. As stated by Slaninova et al. [132], tissue GSH levels are often depleted after short-term oxidant exposures, but elevated after long-term exposures. Moreover, Zhang et al. [133] reported that during a moderate oxidative stress, the GSH levels can increase as an adaptive mechanism by means of an increased synthesis. In addition, GR catalyzes the reduction of GSSG to GSH. In contrast to GPx, this enzyme is involved in the maintenance of GSH in its reduced form and, owing to this, GSH has its antioxidant functions [134,135]. We agree with Spolarics & Wu [136], De Bleser et al. [137], Merad-Saidoune et al. [138], and Łukaszewicz-Hussain & Moniuszko-Jakoniuk [125], who suggested that GPx is responsible for the detoxification of H_2_O_2_ when it is present in low concentrations, whereas CAT plays its role when the GPx pathway reaches saturation with the substrate.

### 4.4. Behavioral Responses

The toxic effect of the pesticides contributed to the observed behavioral responses, even though the concentrations were lower than their MAC-EQS in water. Our results are in agreement with the findings of Viran et al. [79], Başer et al. [139], Singh & Singh [140], Borges et al. [141], and Bab et al. [142], who report not only histological alterations and enzymatic changes after contact with the studied pesticides, but also impaired swimming behavior.

## 5. Conclusions

Even though the common carp is considered to be a fish species relatively resilient to water contamination, the present study increases our knowledge on how concentrations lower than the MAC-EQS could affect its health. Furthermore, in experimental conditions CYP resulted to be more toxic than CPF on the studied biomarkers. Furthermore, CPF in combination with CYP may have a synergistic cumulative effect, which was confirmed in the study of Vardavas et al. [143]. Therefore, further research should be carried out in this area in order to compare the toxic character of these two pesticides, prevent their negative impact, and learn more about the possible effects of long-term exposure to CPF and CYP in common carp.

The results obtained from this experiment could help us to further understand the toxicity of pesticides on non-target organisms and to better serve plant protection practices and environmental safety. Lastly, the results could be used for setting an adequate regulatory framework regarding the presence of priority organic pollutants in biota, which many EU countries still have not done.

## Figures and Tables

**Table 1:** Average results on the basic physicochemical properties of the water treated with chlorpyrifos (CPF) and cypermethrin (CYP) for 50 L tanks (measured at the 24th, 48th, 72nd, and 96th hour).

		Concentration of CPF (µg/L)			Concentration of CYP (µg/L)		
Parameters	Control	0.03	0.05	0.10	0.0002	0.0003	0.0006
Conductivity (μS/cm)	509.33	525.03	513.00	530.03	487.13	529.25	543.75
Dissolved oxygen (mg/L)	9.05	9.10	9.20	9.10	8.83	8.75	9.08
pH	7.73	7.42	7.21	7.03	7.52	7.57	7.50
T (°C)	12.07	11.59	11.68	11.70	11.32	11.25	11.59

**Table 2:** Average results on the concentration of chlorpyrifos (CPF) and cypermethrin (CYP) in the water (µg/L) and fish organs (µg/g wet weight) measured at the 96th hour (mean value ± standard deviation), as well as the calculated bioconcentration factor (BCF).

		**Total Concentration of CPF, µg (for 50 L Tanks)**		
	**Control**	**1.5**	**2.5**	**5**
Water	n.d.	0.76 ± 0.25 ^c^	1.69 ± 0.19 ^b^	2.76 ± 0.17 ^a^
Gills	n.d.	0.202 ± 0.03 ^b^	0.21 ± 0.03 ^b^	0.276 ± 0.61 ^a^
Liver	n.d.	0.184 ± 0.03 ^b^	0.19 ± 0.02 ^b^	0.27 ± 0.06 ^a^
BCF, gills	-	3.76 *	8.05 *	10 *
BCF, liver	-	4.1 *	8.9 *	10.2 *
		**Total Concentration of CYP, µg (for 50 L Tanks)**		
	**Control**	**0.01**	**0.015**	**0.03**
Water	n.d.	0.0009 ± 0.0002 ^c^	0.008 ± 0.001 ^b^	0.017 ± 0.004 ^a^
Gills	n.d.	0.063 ± 0.02 ^a^	0.878 ± 0.025 ^a^	0.092 ± 0.034 ^a^
Liver	n.d.	0.055 ± 0.02 ^b^	0.079 ± 0.029 ^a,b^	0.084 ± 0.025 ^a^
BCF, gills	-	0.14	0.009	1.85
BCF, liver	-	0.02	0.1	2.02 *

^a,b,c^ The values with different letters in the same row are significantly different (Tukey’s test, *p* < 0.05). n.d.: not detectable. The limits of quantitation (LoQ) for CPF in water and fish were set at 0.005 µg/L and 0.15 µg/g and for CYP at 0.0002 µg/L and 0.009 µg/g wet weight, respectively. *—BCF > 2.

**Table 3:** Histological lesions in the gills of common carp after 96-h exposure to chlorpyrifos (CPF) and cypermethrin (CYP).

Reaction Pattern	Functional Unit of the Tissue	Alteration	Importance Factor	Score Value— Concentrations of CPF (μg/L)				Index for Each Group (0.03; 0.05; 0.1 μg/L)
				Control	0.03	0.05	0.10	
Circulatory disturbances	Gills Blood vessels of secondary lamellae	Vasodilation	W_GC1_ = 1	0	0	0	1	I_GC_ = 2 I_GC_ = 2 I_GC_ = 3
	Gills Blood vessels of primary lamellae	Vasodilation	W_GC4_ = 2	0	1	1	1	
Regressive lesions	Gills Epithelium	Degeneration (necrosis)	W_GR1_ = 3	0	0	1	1	I_GR_ = 0 I_GR_ = 3 I_GR_ = 3
Progressive lesions	Gill epithelium (secondary lamellae)	Lamellar lifting	W_GP1_ = 1	0	3	3	3	I_GP_ = 17 I_GP_ = 18 I_GP_ = 22
		Proliferation	W_GP1_ = 2	0	2	2	1	
	Gill epithelium (primary lamellae)	Edema	W_GP2_ = 1	0	1	1	1	
		Proliferation of stratified epithelium	W_GP3_ = 2	0	3	2	2	
		Proliferation of glandular cells	W_GP4_ = 1	0	0	0	0	
		Fusion	W_GP5_ = 3	0	1	2	4	
**Index organ**				**I_C_ = 0**	**I_0.03_ = 19**	**I_0.05_ = 23**	**I_0.1_ = 28**	
**Reaction Pattern**	**Functional Unit of** **the Tissue**	**Alteration**	**Importance Factor**	**Score Value—** **Concentrations of CYP (μg/L)**				**Index for Each Group** **(0.0002; 0.0003; 0.0006 μg/L)**
				**Control**	**0.0002**	**0.0003**	**0.0006**	
Circulatory disturbances	Gills Blood vessels of secondary lamellae	Vasodilation	W_GC1_ = 1	0	1	1	1	I_GC_ = 3 I_GC_ = 3 I_GC_ = 5
	Gills Blood vessels of primary lamellae	Vasodilation	W_GC4_ = 2	0	1	1	2	
Regressive lesions	Gills Epithelium	Degeneration (necrosis)	W_GR1_ = 3	0	1	2	2	I_GR_ = 3 I_GR_ = 6 I_GR_ = 6
Progressive lesions	Gill epithelium (secondary lamellae)	Lamellar lifting	W_GP1_ = 1	0	1	1	1	I_GP_ = 19 I_GP_ = 19 I_GP_ = 19
		Proliferation	W_GP1_ = 2	0	2	2	2	
	Gill epithelium (primary lamellae)	Edema	W_GP2_ = 1	0	1	1	1	
		Proliferation of stratified epithelium	W_GP3_ = 2	0	2	2	2	
		Proliferation of glandular cells	W_GP4_ = 1	0	0	0	0	
		Fusion	W_GP5_ = 3	0	3	3	3	
**Index organ**				**I_C_ = 0**	**I_0.0002_ = 25**	**I_0.0003_ = 28**	**I_0.0006_ = 30**	

**Table 4:** Histological lesions in the liver of common carp after 96-h exposure to chlorpyrifos (CPF) and cypermethrin (CYP).

Reaction Pattern	Functional Unit of the Tissue	Alteration	Importance Factor	Score Value— Concentrations of CPF (μg/L)				Index for Each Group (0.03; 0.05; 0.1 μg/L)
				Control	0.03	0.05	0.1	
Circulatory disturbances	Liver	Hyperemia	W_LC1_ = 1	0	1	2	2	I_LC_ = 1 I_LC_ = 2 I_LC_ = 2
		Intercellular edema		0	0	0	0	
Regressive lesions	Liver	Granular degeneration	W_LR1_ = 1	1	2	3	3	I_LR_ = 8 I_LR_ = 15 I_LR_ = 22
		Deposits (lipids)	W_LR3_ = 1	0	0	1	1	
		Nuclear alterations	W_LR4_ = 2	0	1	1	2	
		Necrosis	W_LR5_ = 3	0	0	1	2	
		Vacuolar degeneration	W_LR6_ = 2	0	2	3	4	
	Interstitial tissue	Architectural and structural alterations	W_LR7_ = 1	0	0	0	0	
		Deposits	W_LR8_ = 1	0	0	0	0	
		Nuclear alterations	W_LR9_ = 2	0	0	0	0	
		Necrosis	W_LR10_ = 3	0	0	0	0	
Progressive lesions	Liver	Hypertrophy	W_LP1_ = 1	0	1	1	2	I_LP_ = 1 I_LP_ = 1 I_LP_ = 2
	Interstitial tissue	Hypertrophy	W_LP2_ = 1	0	0	0	0	
Inflammation	Liver	Activation of RES	W_LI1_ = 1	0	0	0	0	I_LI_ = 0 I_LI_ = 1 I_LI_ = 2
		Infiltration	W_LI2_ = 2	0	1	1	1	
**Index organ**				**I_C_ = 1**	**I_0.03_ = 10**	**I_0.05_ = 19**	**I_0.1_ = 28**	
**Reaction Pattern**	**Functional Unit of** **the Tissue**	**Alteration**	**Importance Factor**	**Score Value—** **Concentrations of CYP (μg/L)**				**Index for Each Group (0.0002; 0.0003; 0.0006 μg/L)**
				**Control**	**0.0002**	**0.0003**	**0.0006**	
Circulatory disturbances	Liver	Hyperemia	W_LC1_ = 1	0	1	3	3	I_LC_ = 1 I_LC_ = 3 I_LC_ = 3
		Intercellular edema		0	0	0	0	
Regressive lesions	Liver	Granular degeneration	W_LR1_ = 1	1	3	2	2	I_LR_ = 11 I_LR_ = 14 I_LR_ = 20
		Deposits (lipids)	W_LR3_ = 1	0	1	1	2	
		Nuclear alterations	W_LR4_ = 2	0	1	1	2	
		Necrosis	W_LR5_ = 3	0	1	1	2	
		Vacuolar degeneration	W_LR6_ = 2	0	1	3	3	
	Interstitial tissue	Architectural and structural alterations	W_LR7_ = 1	0	0	0	0	
		Deposits	W_LR8_ = 1	0	0	0	0	
		Nuclear alterations	W_LR9_ = 2	0	0	0	0	
		Necrosis	W_LR10_ = 3	0	0	0	0	
Progressive lesions	Liver	Hypertrophy	W_LP1_ = 1	0	1	2	2	I_LP_ = 1 I_LP_ = 2 I_LP_ = 2
	Interstitial tissue	Hypertrophy	W_LP2_ = 1	0	0	0	0	
Inflammation	Liver	Activation of RES	W_LI1_ =1	0	0	0	0	I_LI_ = 2 I_LI_ = 4 I_LI_ = 6
		Infiltration	W_LI2_ = 2	0	1	2	3	
**Index organ**				**I_C_ = 1**	**I_0.0002_ = 15**	**I_0.0003_ = 23**	**I_0.0006_ = 31**	

## Data Availability

The data presented in this study are available on request from the corresponding author.
